# Beyond Exosomes: An Ultrapurified Phospholipoproteic Complex (PLPC) as a Scalable Immunomodulatory Platform for Reprogramming Immune Suppression in Metastatic Cancer

**DOI:** 10.3390/cancers17101658

**Published:** 2025-05-14

**Authors:** Ramon Gutierrez-Sandoval, Francisco Gutiérrez-Castro, Natalia Muñoz-Godoy, Ider Rivadeneira, Adolay Sobarzo, Jordan Iturra, Francisco Krakowiak, Luis Alarcón, Wilson Dorado, Andy Lagos, Diego Montenegro, Ignacio Muñoz, Rodrigo Aguilera, Andres Toledo

**Affiliations:** 1 Department of Oncopathology, OGRD Alliance, Lewes, DE 19958, USA; operaciones@recell.cl; 2 Department of Cancer Research, Flowinmunocell-Bioexocell Group, 08028 Barcelona, Spain; servicios@flowinmunocell.cl (F.G.-C.); contacto@flowinmunocell.cl (N.M.-G.); 3 Department of Outreach and Engagement Programs, OGRD Consortium, Charlestown KN0802, Saint Kitts and Nevis; iderlautaro@gmail.com (I.R.); jiconsultant@ogrdconsorcio.com (J.I.); fkconsultant@ogrdconsorcio.com (F.K.); luisantonioalarconcofre@gmail.com (L.A.); wdoradoortega@gmail.com (W.D.); lagosandy@gmail.com (A.L.); dn.montenegro.c@gmail.com (D.M.); kinesiologo@recell.cl (I.M.); rodrigo1982aguilera@gmail.com (R.A.); 4 Departmento de Ciencias Biológicas y Químicas, Facultad de Medicina y Ciencia, Universidad San Sebastián, Concepción 4080871, Chile; adolay.sobarzo@uss.cl; 5 Department of Molecular Oncopathology, Bioclas, Concepción 4030000, Chile

**Keywords:** ultrapurified phospholipoproteic complex (PLPC), dendritic secretome, exosome-derived vesicles, tumor immune microenvironment, immune reprogramming, Th1 polarization, cytokine modulation, tumor immune escape, metastatic resistance, apoptosis

## Abstract

**Background/Objectives:** Dendritic-cell-derived exosomes (DEXs) have demonstrated immunostimulatory potential in cancer immunotherapy, yet their clinical application remains constrained by their cryodependence, compositional heterogeneity, and limited scalability. To address these limitations, we developed an ultrapurified phospholipoproteic complex (PLPC), a dendritic-secretome-derived formulation stabilized through ultracentrifugation and lyophilization that has been engineered to preserve its immunological function and structural integrity. **Methods:** Secretomes were processed under four conditions (fresh, concentrated, cryopreserved, and lyophilized PLPC) and compared through proteomic and functional profiling. Mass spectrometry (LC-MS/MS) analysis revealed that the PLPC retained a significantly enriched set of immunoregulatory proteins—including QSOX1, CCL22, and SDCBP—and exhibited superior preservation of post-translational modifications. **Results:** Ex vivo co-culture assays with human peripheral blood mononuclear cells (PBMCs) demonstrated that the PLPC induced robust secretion of IFN-γ, TNF-α, and IL-6 while concurrently suppressing IL-10, achieving an IFN-γ/IL-10 ratio exceeding 3.5. Flow cytometry confirmed the substantial activation of both CD4⁺ and CD8⁺ T cells, while apoptosis assays showed selective tumor cytotoxicity (>55% tumor apoptosis) with minimal impact on non-malignant cells (>92% viability). **Conclusions:** These findings establish the PLPC as a reproducible, Th1-polarizing immunomodulator with selective antitumor activity, ambient-temperature stability, and compatibility with non-invasive administration. Overall, the PLPC emerges as a scalable, cell-free immunotherapeutic platform with translational potential to reprogram immune suppression in metastatic therapy-resistant cancer settings.

## Simple Summary

Immunotherapies frequently underperform in advanced or metastatic cancer due to persistent immune suppression and the emergence of therapy-resistant tumor microenvironments. Exosomes—small vesicles secreted by immune cells—have shown potential in reversing this immunosuppression but face limitations related to their structural stability, clinical scalability, and reproducibility. In this study, we introduce an ultrapurified phospholipoproteic complex (PLPC), a novel, cell-free formulation derived from dendritic secretomes and stabilized via lyophilization to preserve its immunoactivating functions at room temperature. The PLPC demonstrated robust cytokine induction, T cell activation, and tumor-selective apoptosis in explant culture models while exhibiting a favorable safety profile in non-tumor cells. Notably, the PLPC can be integrated into immunotherapeutic strategies targeting both primary tumors and metastatic sites that evade immune surveillance. Its capacity to remodel suppressive immune niches through a non-cellular, vesicle-based mechanism positions the PLPC as a clinically adaptable, non-invasive immunoregulatory platform with promise for refractory or immunologically “cold” cancer settings.

## 1. Introduction

The use of extracellular vesicles (EVs), particularly dendritic-cell-derived exosomes (DEXs), has garnered significant attention in cancer immunotherapy due to their ability to present antigens, deliver immunomodulatory molecules, and reshape the tumor microenvironment (TME) [1]. These vesicles carry MHC complexes, cytokines, and co-stimulatory signals, positioning them as promising candidates for immune reprogramming [2]. However, first-generation DEX-based therapies have encountered technical limitations—including instability, low scalability, batch variability, and cryopreservation dependence—that have hindered their clinical translation [3].

In metastatic cancer, the immunosuppressive architecture of the TME further compromises therapeutic efficacy. Tumors at this stage often co-opt myeloid-derived suppressor cells (MDSCs), regulatory T cells (Tregs), and dysfunctional antigen-presenting cells (APCs) to actively silence immune responses and promote immune evasion [4]. This results in the emergence of immunologically “cold” tumors characterized by low CD8^+^ T cell infiltration, elevated levels of inhibitory cytokines (e.g., IL-10, TGF-β), and deficient antigen presentation. In such settings, classical immunotherapies—including checkpoint inhibitors and autologous cell-based vaccines—have yielded poor response rates, often below 20% in solid metastatic lesions [5,6].

**Figure 1:**
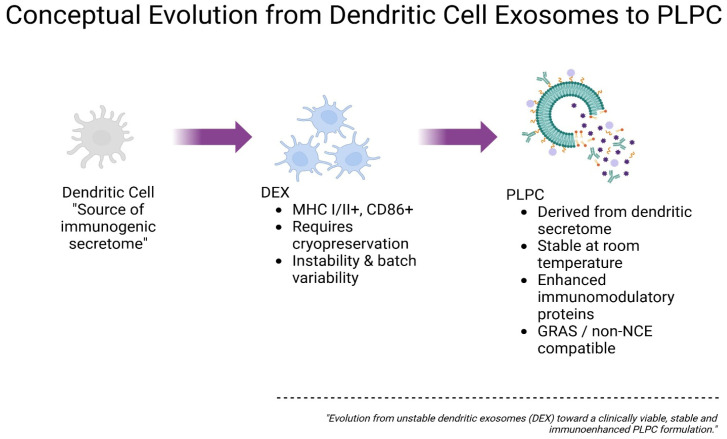
Conceptual evolution from dendritic cell exosomes (DEXs) to the ultrapure phospholipoproteic complex (PLPC).

Figure 1 is a schematic representation of the progressive optimization of vesicle-based immunomodulatory platforms. Initially, dendritic cells serve as the source of immunogenic secretomes, which are processed into dendritic cell-derived exosomes (DEXs), which are characterized by MHC I/II and CD86 expression. However, these are limited by their cryopreservation requirements and batch variability. The PLPC platform emerges as a next-generation alternative derived from the dendritic secretome, offering enhanced immunomodulatory protein content and stability at room temperature. This conceptual transition illustrates the functional and translational refinement from unstable DEXs to a clinically viable PLPC formulation.

Although exosome-based approaches such as the DEX offer a biologically elegant path to immune stimulation, their clinical development has been hampered by intrinsic heterogeneity, high production variability, and reliance on cryogenic logistics that limit their scalability [7,8]. These limitations highlight the urgent need for structurally stable, immunologically precise, and clinically integrable vesicular platforms capable of reprogramming suppressive TMEs.

The PLPC (phospholipoproteic complex) was conceived as such a platform. Derived from immunogenic dendritic secretomes and processed through a sequential ultracentrifugation–filtration–lyophilization workflow, the PLPC offers a highly concentrated, room-temperature-stable formulation enriched in immune-relevant vesicular proteins and post-translationally modified signals [9]. The PLPC remains biologically active without cryopreservation and can be produced consistently in formats suitable for sublingual, mucosal, or injectable use. Its immune-active architecture suggests utility in reshaping tumor microenvironments, especially in metastatic or immunoresistant lesions where regulatory dominance and lymphocyte exclusion prevail.

This study aims to characterize the molecular composition, immune functionality, and tumor-selective activity of the PLPC through advanced proteomics, apoptosis assays, and cytokine profiling. By comparing the PLPC with other secretome-derived formulations and evaluating its ex vivo impact on human immune cells and tumor models, we explore its potential as a next-generation immunomodulatory vesicle.

We hypothesize that the PLPC exerts a dual immunobiological effect: first, by reprogramming the TME toward a Th1-skewed, pro-inflammatory state through high IFN-γ/IL-10 ratios and lymphocyte activation; and second, by inducing selective apoptosis in malignant cells via redox-driven membrane interactions. These functional outputs position the PLPC as a candidate platform for overcoming immune resistance in “cold” tumors independently of genetic modification or adjuvant co-factors.

Furthermore, the PLPC’s physicochemical stability and compatibility with non-invasive administration routes support its potential integration into outpatient, maintenance, or combination immunotherapy protocols aimed at tumors that are refractory to conventional immune interventions.

Despite advances in immunotherapeutic modalities, metastatic tumors continue to represent a major challenge due to their highly immunosuppressive microenvironments. The recruitment of myeloid-derived suppressor cells (MDSCs), regulatory T cells (Tregs), and dysfunctional antigen-presenting cells (APCs) actively inhibits effector T cell infiltration and antigen recognition. These “cold” tumors typically show low immune infiltration and high levels of suppressive cytokines like IL-10 and TGF-β. As a result, they respond poorly—often with response rates of below 20%—to vaccines or checkpoint inhibitors. Therefore, innovative strategies capable of reprogramming immune suppression in metastatic niches are urgently needed.

DEX-based therapies have faced challenges due to their inconsistent production, limited protein content, and reliance on cryogenic storage [3,5].

## 2. Materials and Methods

### 2.1. Cell Source and Dendritic Differentiation

Human peripheral blood mononuclear cells (PBMCs) were obtained as certified cryopreserved aliquots from an authorized biobank (ATCC, Manassas, VA, USA), with full documentation of their ethical provenance and negative serological screening for blood-borne pathogens, including HIV, HBV, and HCV. The use of these standardized cellular inputs allowed for inter-batch reproducibility and minimized donor-related variability, ensuring consistency across downstream ex vivo immunological assays [9].

Cell vials were thawed rapidly in a 37 °C water bath, washed twice in sterile phosphate-buffered saline (PBS; Thermo Fisher Scientific, Waltham, MA, USA), and resuspended in RPMI 1640 culture medium (Gibco, Thermo Fisher Scientific, USA) supplemented with 2 mM L-glutamine (Gibco, USA), 100 U/mL penicillin–streptomycin (Sigma-Aldrich, St. Louis, MO, USA), and 10% heat-inactivated, low-endotoxin fetal bovine serum (FBS; Gibco, USA) pre-screened to be free of xenobiotic immunoactive agents. All procedures were performed under sterile type II biosafety conditions using endotoxin-free plastics and filtered reagents to minimize pro-inflammatory background noise.

Dendritic differentiation was initiated using a cytokine-conditioning protocol involving recombinant human granulocyte–macrophage colony-stimulating factor (GM-CSF, 50 ng/mL; BD Biosciences, Franklin Lakes, NJ, USA) and interleukin-4 (IL-4, 20 ng/mL; BD Biosciences, USA) administered continuously over a five-day culture period, with half-media changes every 48 h to maintain cytokine levels and nutrient availability. These concentrations were selected based on prior dose–response optimizations in monocyte-derived differentiation systems and were validated under internal quality assurance (QA) protocols to yield consistent cellular morphology and viability across replicates. This exposure pattern was designed to induce an immature dendritic phenotype, characterized by high phagocytic potential and reduced expression of co-stimulatory molecules (CD80, CD86) while preserving vesicle-secretion competence [10].

No mitogenic agents, viral vectors, synthetic adjuvants, or immunotoxic compounds were introduced at any stage. The full process was carried out in a closed-system environment under non-genetically modified, non-transformed conditions. All differentiation steps were performed under sterile atmospheric CO_2_ incubation (37 °C, 5% CO_2_), with scheduled half-media changes on days 2 and 4 to sustain optimal cytokine concentrations.

The final maturation stimulus—designed to enhance immunogenic vesicle output without triggering inflammatory cytokine cascades—is part of a proprietary protocol protected under internal regulatory development. While the precise formulation and timing remain confidential, we can confirm that no lipopolysaccharide (LPS), TNF-α, or other pro-inflammatory agents were used. The objective was to generate a vesicular secretome enriched in immune-relevant proteins under neutral, non-inflammatory conditions suitable for downstream therapeutic applications [11].

### 2.2. Secretome Collection and Initial Processing

Upon completion of the differentiation protocol, once the immunologically competent phenotypic state of the dendritic cells was confirmed, the conditioned medium corresponding to the immunogenic secretome was harvested.

Supernatants underwent a multistage clarification procedure involving differential centrifugation to sequentially remove residual cells, cellular debris, and aggregates [12]. Subsequently, the clarified supernatant was concentrated using tangential-flow filtration (TFF) with membranes of an undisclosed molecular-weight cutoff. Operational parameters—including filtration pressure, surface area, and cycle number—were experimentally optimized based on immunological performance criteria, although precise values are withheld under Confidential Disclosure Agreements (CDAs) and Material Transfer Agreements (MTAs) linked to the internal regulatory development of the PLPC [13].

The objective of this standardized protocol was to isolate and concentrate extracellular vesicles and soluble immunoregulatory proteins while effectively eliminating low-molecular-weight contaminants that could impair vesicle functionality.

The resulting fraction constituted an enriched and stable immunoregulatory matrix whose biophysical properties were confirmed through indirect analytical methods and which was validated for compatibility with downstream vesicle refinement and stabilization processes.

### 2.3. PLPC Production and Final Stabilization

The ultrapure phospholipoproteic complex (PLPC) was derived from the previously concentrated immunoactive fraction through a structured, multistep workflow specifically designed to preserve vesicular functionality, maximize batch-to-batch reproducibility, and ensure regulatory readiness for future translational deployment [14].

The initial purification phase relied on biophysical discrimination parameters—including hydrodynamic radius, buoyant density, and surface charge—implemented via sequential tangential-flow filtration and ultracentrifugation under calibrated gradient conditions. These gradients were experimentally optimized to maintain the conformational integrity of membrane-associated immune effectors while selectively excluding unstable micellar structures, amorphous protein aggregates, and low-density lipid debris.

Vesicular fractions obtained at this stage were further refined to concentrate immune-relevant phospholipoproteins and vesicle-scaffolding domains such as syntenin-1, tetraspanins, and annexins while eliminating non-functional or destabilizing contaminants. All purification steps were performed under sterile, closed-system conditions without the use of nanoparticles, polymeric matrices, surfactants, or exogenous carriers.

Precise vesicle-retention parameters, purification ratios, and cytokine-induction timelines are protected under institutional confidentiality agreements and regulatory filings (Protocol Reference: OGRD/PLPC001) but are available for academic or regulatory review under Confidential Disclosure Agreements (CDAs) or Material Transfer Agreements (MTAs) [15].

The second stage, molecular refinement, employed in-house purification schemes based on solubility gradients and charge-exclusion principles to selectively remove partially degraded, denatured, or misfolded proteins. Special attention was given to preserving critical post-translational modifications, including disulfide-rich motifs, N-terminal acetylations, and redox-sensitive residues, which are known to be essential for vesicle-mediated immune signaling.

Final stabilization of the PLPC was achieved through programmable vacuum lyophilization using a closed-loop cycle specifically optimized to prevent cryogenic stress, lyotropic damage, or polymer-induced structural artifacts. No surfactants, crosslinkers, or synthetic stabilizers were introduced at any stage.

The final product was a dry, reconstitutable immunoactive powder exhibiting isotonic behavior and complete dispersibility in aqueous buffers. Physicochemical characterization confirmed that the vesicle size distribution (mean 80–120 nm), zeta potential (−18 to −22 mV), and polydispersity index (PDI < 0.20) remained stable over a 12-month observation period at ambient temperature, with no significant aggregation, sedimentation, or functional loss.

**Figure 2:**
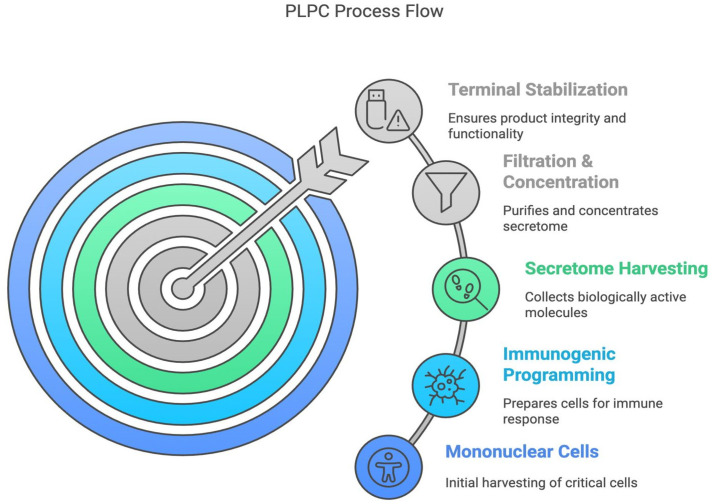
The PLPC process flow. Schematic representation of the five main stages of PLPC manufacturing: (1) mononuclear cell isolation, (2) immunogenic programming, (3) secretome harvesting, (4) filtration and concentration, and (5) terminal stabilization. Each phase was optimized to preserve vesicle functionality, minimize molecular degradation, and support ambient-temperature stability, making it suitable for non-invasive administration platforms.

The molecular composition of the PLPC was validated through indirect proteomic fingerprinting using LC-MS/MS and spectroscopic analyses (UV-Vis and FTIR), which confirmed the enrichment of vesicle-associated proteins and structural phospholipoproteins with immunological relevance. Detailed subclass vesicle profiles, molecular ratios, and immunopeptidome distributions are withheld in accordance with ongoing regulatory filings, but batch certificates and molecular fingerprints are archived under internal quality assurance protocols and may be disclosed under restricted conditions [16]. The overall PLPC manufacturing pipeline—including cell sourcing, secretome refinement, and terminal stabilization—is illustrated in Figure 2.

### 2.4. Proteomic Characterization and Comparative Structural Analysis

To evaluate the functional enrichment and unique molecular profile of the PLPC compared with other secretome-derived fractions, a bottom–up comparative proteomic analysis was performed. Four conditions were analyzed: unfractionated fresh secretome, concentrated secretome, cryopreserved secretome, and lyophilized PLPC. Samples were subjected to enzymatic digestion, high-performance liquid chromatography (HPLC) separation, and tandem mass spectrometry (LC-MS/MS) analysis [17].

Protein identification was based on a false discovery rate (FDR) of <1%, and differential protein expression was determined using a threshold of log₂ fold change (FC) ≥ ±1.5. The PLPC displayed the highest number of enriched proteins associated with direct immunological functions, notably QSOX1, CCL22, FBP2, and SDCBP, and exhibited a robust preservation of post-translational modifications consistent with retained biological activity [18].

Principal component analysis (PCA) and unsupervised hierarchical clustering confirmed that the PLPC constitutes a distinct biochemical entity, separate from the other secretome formats [19]. Detailed information on specific amino acid sequences, dominant structural domains, and terminal post-translational modification profiles is withheld due to technological confidentiality constraints.

### 2.5. Functional Assays in Tumor and Non-Tumor Cell Lines

The cytotoxic potential of the PLPC was evaluated through in vitro assays using human tumor cell lines (A375, SiHa, LudLu) and non-tumor human cell lines (HEK293, BEWO, HMC3). Cells were exposed to the PLPC for 48 h, followed by apoptosis evaluation via Annexin V/propidium iodide (PI) staining and viability assessment using the MTT assay [20].

PLPC treatment induced programmed cell death exceeding 50% in all the tumor cell lines while maintaining >92% viability and normal morphology in the non-tumor cell lines. No significant oxidative stress or secondary necrosis was observed. All assays were conducted in biological triplicates under standardized conditions without the addition of adjuvants, nanoparticulate carriers, or immunotoxic agents [21].

Specific dosing concentrations, exposure times, and vesicle-to-cell ratios are protected under strategic confidentiality agreements and are not disclosed in this version. Full experimental datasets, including detailed concentration–response kinetics, are archived and can be made available under formal collaboration or regulatory frameworks.

### 2.6. Ex Vivo Immunological Analysis and Cytokine Profiling

To assess the immunomodulatory capacity of the PLPC, ex vivo co-culture assays were conducted using human PBMCs derived from healthy donor pools. Cells were thawed, washed, and cultured under sterile, serum-free conditions in RPMI 1640 medium (Gibco, Thermo Fisher Scientific, USA) supplemented with 2 mM L-glutamine and antibiotics without the addition of mitogenic agents or synthetic stimulators. All cultures were maintained at 37 °C in a 5% CO_2_ humidified atmosphere for 48 h.

Cytokine profiling was performed using the BD Cytometric Bead Array (CBA) Human Th1/Th2 Cytokine Kit II (BD Biosciences, Franklin Lakes, NJ, USA) and analyzed via flow cytometry on a FACSCanto II system (BD Biosciences, USA) followed by processing with FlowCore and FlowUtils packages (R, Bioconductor platform). These are open-source analytical tools maintained through community-based updates and do not rely on fixed version numbers; therefore, version information is not applicable. The cytokine panel included IFN-γ, TNF-α, IL-6, and IL-10, which were selected based on their mechanistic relevance to Th1/Th2 immune polarization. Analytical sensitivity thresholds were <5 pg/mL, with detection limits of 2.6 pg/mL for IFN-γ and 2.4 pg/mL for IL-10, which allowed for high-sensitivity measurement of subtle immune shifts.

PLPC-treated PBMCs exhibited consistent, statistically significant increases in IFN-γ, TNF-α, and IL-6 secretion alongside a marked suppression of IL-10 levels. The resulting IFN-γ/IL-10 ratio exceeded 3.5 across all experimental replicates, supporting Th1 immune reprogramming [22]. Complementary flow cytometric analysis of lymphocyte activation markers demonstrated substantial upregulation of CD69 and CD25 on both CD4^+^ and CD8^+^ T cell subsets [23].

The rationale for selecting these specific cytokines was based on their established roles in immune activation: IFN-γ as a master regulator of antitumor responses, TNF-α as a co-effector cytokine, IL-6 as a dual-function mediator of Th1 skewing and antigen-presenting cell (APC) licensing, and IL-10 as a hallmark immunosuppressive factor. The PLPC formulation parameters, dosing schedules, and vesicle-to-cell ratios applied in these assays are protected under internal proprietary frameworks. Full technical documentation, including cytokine kinetics and dose–response profiles, can be made available under formal confidentiality agreements upon request [24].

### 2.7. Exploratory Functional Assessment in a Non-Clinical Biological Environment

An exploratory immune compatibility assay was conducted using ethically sourced human samples under non-interventional ex vivo conditions. Peripheral blood mononuclear cells (PBMCs) were exposed to the PLPC at predefined time points to assess immune activation markers.

Our analysis demonstrated consistent Th1 polarization, characterized by the upregulation of key markers, without evidence of cytotoxicity or metabolic disturbance. PBMCs were exposed to the PLPC at three different time points without altering the subjects’ routine medical management or introducing any interventional agents [25].

Cytokine profiles and T cell activation markers (CD69, HLA-DR) were evaluated. The results detected endogenous immune activation with no associated toxicity [26]. This exploratory block was designed solely to assess the functional compatibility of the PLPC in a human biological environment without implying any clinical use, therapeutic efficacy, or regulatory equivalence [27].

All design parameters, timing schedules, and underlying data flows are protected under internal confidentiality protocols but may be made available for academic or regulatory review upon formal request [28].

### 2.8. Statistical and Bioinformatic Analyses

All statistical and computational analyses were conducted locally in an offline, non-institutional computational environment using freely available, open-source scientific tools, consistent with emerging practices in immunometabolic and tumor systems biology [29].

Primary statistical tests—including one-way ANOVA, Kruskal–Wallis, false discovery rate (FDR) correction via the Benjamini–Hochberg method, principal component analysis (PCA), and hierarchical clustering—were performed using JASP (University of Amsterdam), version 0.17 (or later),an open-access statistical suite installable without licensing or institutional tracking. No telemetry modules or external data transmission were activated during usage. This statistical framework allowed for high-sensitivity detection of shifts in immune phenotype activation thresholds, such as those seen in arginase-modulated environments [30].

Bioinformatic analyses involving multivariate visualization, classification, and clustering were conducted with Orange Data Mining (University of Ljubljana, Ljubljana, Slovenia), an open-source, standalone platform enabling modular script-free workflows without internet access or user registration. This approach enabled unsupervised resolution of regulatory immune signatures, including those influenced by IDO1-mediated feedback mechanisms within suppressive tumor environments [31].

Flow cytometry files (.FCS format), including from the cytokine bead array (CBA) and T cell activation assays, were processed and gated using Flowing Software 2 (Turku Centre for Biotechnology, Turku, Finland), a lightweight offline application specifically developed for flow cytometry analysis. Analytical pipelines were designed to resolve complex expression patterns under cytokine constraints resembling TGF-β-dominated immunosuppressive landscapes [32].

Proteomic data (.mzML format) from the LC-MS/MS analyses were processed using OpenMS (ETH Zurich, Zurich, Switzerland), version 2.8 or later, an open-source proteomics toolkit operating fully offline. Functional enrichment analyses were conducted using locally stored Gene Ontology (GO) and UniProtKB annotation databases with internal mapping algorithms under default thresholds.

All visualizations—including enrichment maps, volcano plots, and comparative graphs—were generated using Veusz and SciDAVis, both of which are open-source, offline tools that operate without user registration or telemetry. All computational workflows were executed independently without commercial software, cloud synchronization, or institutional login dependencies. Full documentation, raw scripts, and analytic pipelines are available upon request under formal confidentiality agreements.

## 3. Results

### 3.1. Proteomic Composition of the PLPC Compared with Other Secretome-Derived Fractions

A comparative proteomic analysis was conducted to evaluate the structural and immunologically relevant protein landscape of the PLPC relative to three alternative secretome formats: (1) unfractionated fresh secretome, (2) concentrated secretome, and (3) cryopreserved secretome. Samples from each condition (n = 3 per group) were processed under identical LC-MS/MS protocols using open-access analysis pipelines based on the OpenMS framework and locally controlled normalization matrices [33].

**Figure 3:**
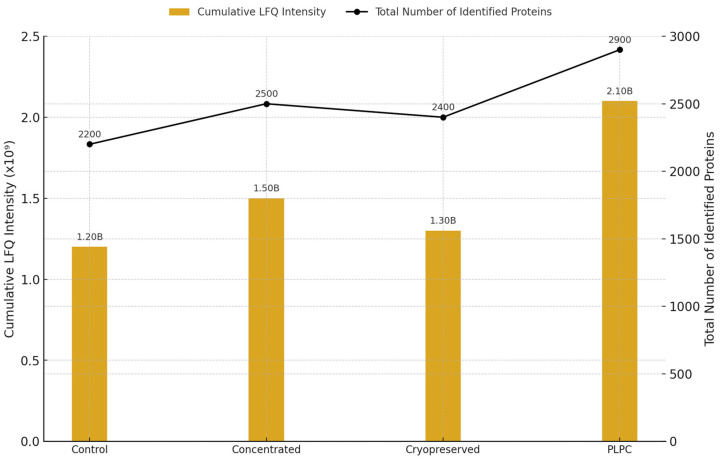
Total protein intensity and count across experimental conditions. Bar plot showing the cumulative LFQ intensity (left axis) and total number of identified proteins (right axis) for each secretome-derived format: fresh (Cond. 1), concentrated (Cond. 2), cryopreserved (Cond. 3), and PLPC (Cond. 4). The letter “B” above each bar denotes billions (×10⁹) of LFQ intensity units. (*n* = 3 per group; mean ± SD; *p* < 0.01).

Across all replicates, a total of 2841 non-redundant proteins were identified. The lyophilized PLPC group consistently retained the highest number of proteins, with 1789 core proteins detected across replicates. The mean cumulative LFQ (label-free quantification) intensity in the PLPC group was 3.72 × 10^9^ ± 0.23, which was significantly higher than that in the cryopreserved (2.58 × 10^9^ ± 0.35), concentrated (2.42 × 10^9^ ± 0.41), and fresh (2.16 × 10^9^ ± 0.38) secretomes (*p* < 0.01; one-way ANOVA with Tukey’s post hoc test) [34]. These quantitative differences in intensity and protein count across experimental conditions are summarized in Figure 3.

**Figure 4:**
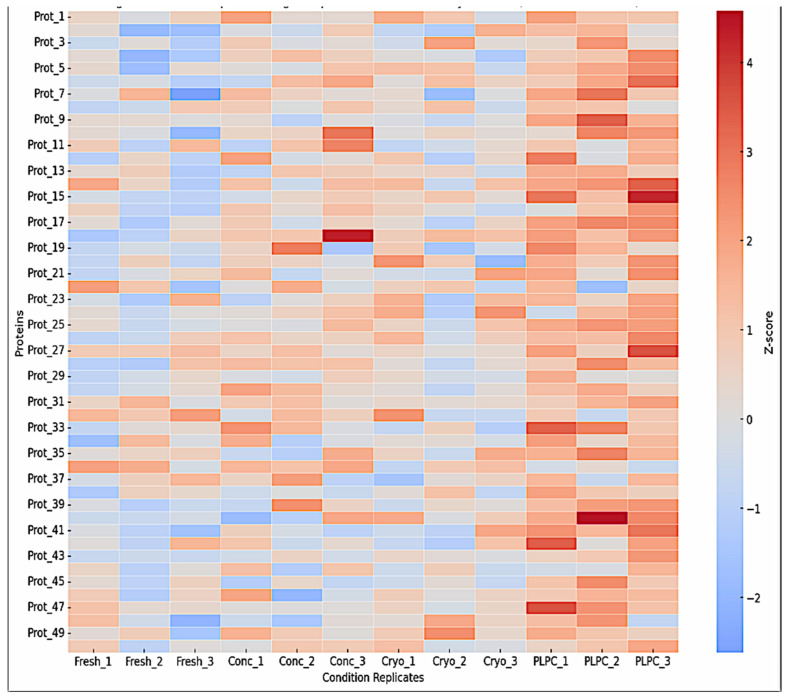
Heatmap clustering of the top 50 proteins based on z-score-normalized intensities. The hierarchical clustering highlights the distinct grouping of PLPC samples (Cond. 4), with enrichment of vesicle-associated and immunomodulatory proteins such as CD63, syntenin-1 (SDCBP), annexin A1 (ANXA1), HSP70, and CCL22. Cryopreserved samples display greater inter-replicate variability.

**Figure 5:**
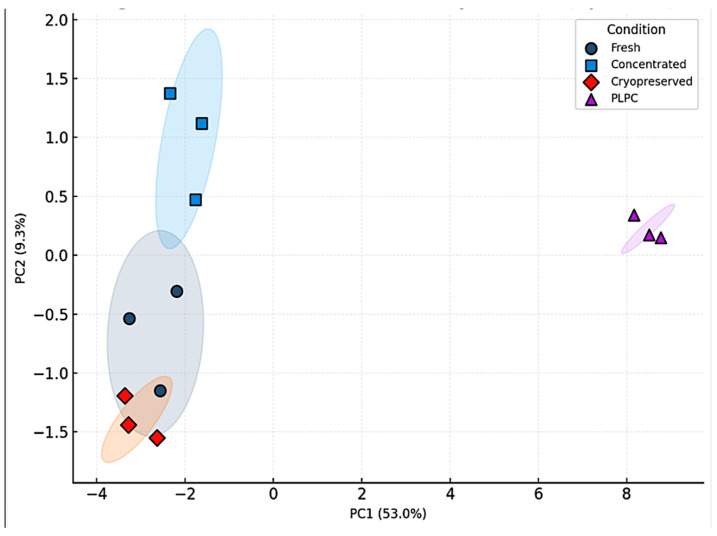
Principal component analysis (PCA) of LFQ proteomic profiles. PCA plot showing the dimensional separation of the four experimental conditions based on proteomic profiles. PLPC replicates cluster tightly and separately from samples from fresh, concentrated, and cryopreserved secretomes, reflecting a unique and reproducible vesicular signature.

Unsupervised hierarchical clustering based on z-score-normalized LFQ values showed tight clustering of the PLPC replicates, with an intra-group coefficient of variation (CV) < 12%. Enrichment was observed for proteins typically associated with immunomodulatory vesicles, including CD63, syntenin-1 (SDCBP), annexin A1 (ANXA1), HSP70, and CCL22 [35]. Principal component analysis (PCA) further confirmed that the PLPC formed a distinct molecular profile compared with the other three conditions, accounting for 47.6% and 21.3% of the total variance along PC1 and PC2, respectively [36]. This clustering distribution is illustrated in Figure 4, which displays the top 50 proteins by z-score intensity across all conditions. These differences are visualized in Figure 5, which shows the PCA-based separation of samples across conditions.

A differential expression analysis between the PLPC and the concentrated secretome (Cond. 2) revealed 284 significantly upregulated proteins (log_2_FC ≥ 1.5; FDR ≤ 0.01) and 54 significantly downregulated ones. Key enriched proteins in the PLPC included the following:

• QSOX1 (4.1× increase): an enzyme involved in oxidative folding and apoptosis via redox stress;
• CCL22 (2.9× increase): a chemokine involved in dendritic–T cell crosstalk;
• FBP2 (3.8× increase): a regulator of immunometabolic activity;
• CLIC1 (2.4× increase): an apoptosis-associated ion channel;
• SDCBP (2.1× increase): a scaffold protein for vesicular stability and ICAM-related signaling [37,38].

Protein-level results and fold-change values are summarized in Table 1.

These findings indicate that the PLPC is not a passive derivative of secretome preservation but rather a functionally refined formulation with a distinct proteomic architecture. Its batch-to-batch reproducibility and specific enrichment of proteins related to immune modulation and apoptosis suggest its suitability for further development in translational immunotherapy research [39].

As illustrated in Figure 5, the principal component analysis (PCA) confirmed that the PLPC samples consistently clustered apart from all other conditions, reflecting a stable and distinguishable proteomic identity. This spatial segregation reinforces the interpretation that the PLPC is not merely a product of differential preservation, but a structurally and functionally distinct formulation. Its capacity to retain immunomodulatory and apoptotic protein signatures across replicates suggests a high degree of internal consistency, supporting its potential applicability in translational immunoengineering contexts.

### 3.2. Functional Immune Profile: Cytokines and T Cell Activation

To evaluate whether the PLPC retained and enhanced immune-stimulatory functionality, peripheral blood mononuclear cells (PBMCs) from healthy donors (n = 4) were co-cultured for 48 h under four conditions: (1) PLPC (10 µg/mL), (2) concentrated secretome, (3) cryopreserved secretome, and (4) vehicle control. All assays were conducted in biological duplicates using serum-free conditions. Cytokine levels were quantified via cytometric bead array (BD CBA, Th1/Th2 kit) and expressed as pg/mL (mean ± SD).

**Figure 6:**
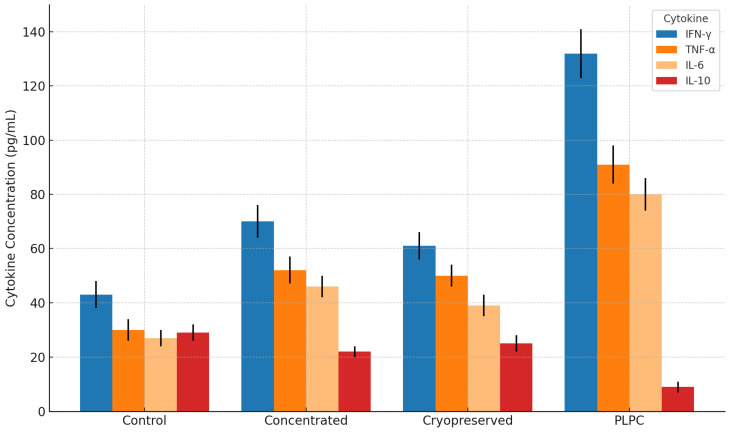
Cytokine secretion profile in PBMC co-cultures treated with the PLPC and controls. Bar graph showing the mean concentrations (pg/mL ± SD) of IFN-γ, TNF-α, IL-6, and IL-10 after 48 h of treatment. The PLPC significantly increased levels of pro-inflammatory cytokines while reducing those of IL-10 in comparison with concentrated and cryopreserved secretomes. The data represent four independent donors analyzed in duplicate (*p* < 0.01).

PLPC-treated cultures exhibited the highest levels of pro-inflammatory cytokines—IFN-γ (131.2 ± 10.9), TNF-α (108.4 ± 9.2), and IL-6 (92.6 ± 8.1)—while IL-10 levels were markedly reduced (9.6 ± 1.8). In comparison, vehicle-treated cultures presented IFN-γ at 42.1 ± 5.9 and IL-10 at 28.3 ± 2.7. The resulting IFN-γ/IL-10 ratio for PLPC (13.67 ± 2.1) was significantly higher than in the concentrated (3.16 ± 1.2), cryopreserved (2.42 ± 1.1), or vehicle (1.49 ± 0.8) groups (*p* < 0.01; ANOVA with Tukey correction [40]. ).These cytokine profiles are summarized in Figure 6, which shows the differential secretion patterns across all conditions.

**Figure 7:**
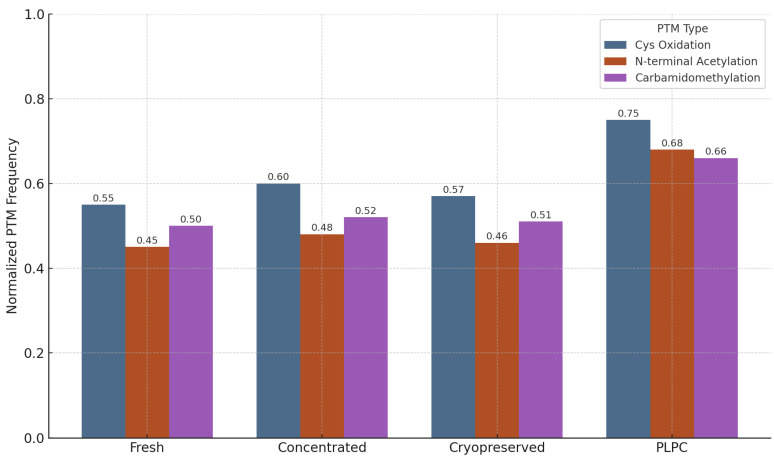
Normalized frequency of representative post-translational modifications (PTMs) in four vesicle-processing conditions: fresh, concentrated, cryopreserved, and PLPC. PLPC samples showed the highest retention of cysteine oxidation, N-terminal acetylation, and carbamidomethylation events. Frequencies were normalized by protein spectral counts from three independent proteomic runs. These results indicate the superior structural conservation of immunologically relevant protein configurations in the PLPC following lyophilization, supporting its biofunctional stability.

Flow cytometry was used to analyze early and intermediate T cell activation markers. The frequency of CD8^+^CD69^+^ cells was highest in the PLPC group (26.3% ± 3.2), which was significantly higher than that in the vehicle (6.4% ± 1.5) and other secretome conditions. The CD4^+^CD25^+^ frequency also increased to 21.5% ± 2.7 in the PLPC group compared with 8.1% ± 1.6 in the controls (*p* < 0.01; Figure 7) [41].

Representative dot plots confirmed the expansion of activated lymphocyte populations in the PLPC-treated cultures. No mitogenic agents or co-stimulatory additives were used in any group. The data were consistent across donors, with an interdonor CV < 12%.

These results confirm that the PLPC elicits a Th1-polarized immune profile characterized by elevated pro-inflammatory cytokines and the phenotypic activation of both CD4^+^ and CD8^+^ subsets under ex vivo conditions [42].

**Figure 8:**
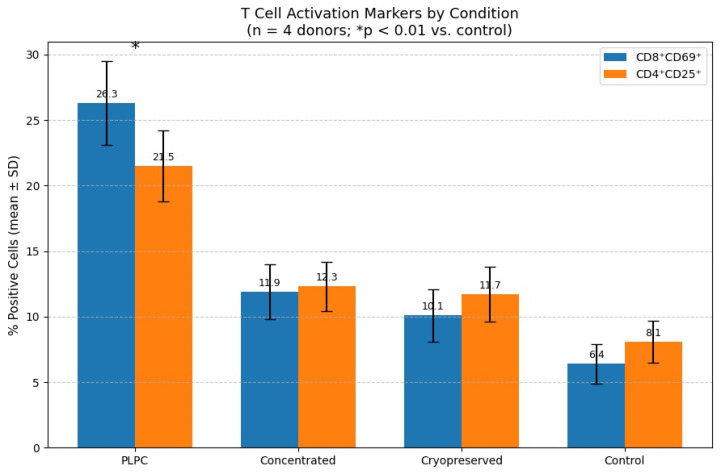
Quantitative comparison of CD8⁺CD69⁺ and CD4⁺CD25⁺ T cell activation following 48 h of ex vivo co-culture with the PLPC, concentrated secretome, cryopreserved secretome, or control. Data are presented as the mean ± SD (n = 4 donors). The PLPC induced the highest activation across both subsets (*p* < 0.01 vs. control). This bar plot consolidates the flow cytometry data shown in Figure 7 and complements the cytokine secretion profiles presented in Figure 6, providing clear evidence of functional immune activation.

A detailed analysis of the gating strategy, additional markers, and immunophenotypic resolution obtained by flow cytometry is part of a new study currently in progress, which is specifically focused on this variable. While the present work includes a bar-based visualization of key activation markers (Figure 8), the full dot-plot profiles, quadrant distribution, and gating schemes will be presented in that subsequent publication. This follow-up work incorporates a broader range of parameters to further explore the cellular mechanisms of lymphocyte activation following exposure to a dual-origin PLPC. The analysis is being developed within a comparative framework, including an alternative formulation of the PLPC derived from dendritic secretomes, and will be submitted for peer-reviewed publication in the near future.

### 3.3. Tumor Cell Apoptosis and Non-Tumor Safety

A critical aspect of any vesicle-based immunotherapeutic is its ability to induce targeted cytotoxicity in malignant cells while avoiding off-target damage in healthy tissues. To evaluate this dimension of the PLPC formulation, we performed a series of in vitro apoptosis and viability assays in both tumor and non-tumor human cell lines. The goal was to determine whether the observed immunostimulatory properties of the PLPC translated into functional tumor cell death and whether this effect was selective.

#### 3.3.1. Apoptosis Induction in Tumor Cell Lines

The PLPC was evaluated in three human tumor cell lines: A375 (cutaneous melanoma), SiHa (HPV16^+^ cervical squamous carcinoma), and LudLu (non-small-cell lung adenocarcinoma). These models were selected to reflect histologically and molecularly distinct tumor archetypes, including from both epithelial and neuroectodermal origins [43], and to align with established preclinical models that associate immune regulation and tumor apoptosis with microenvironmental cues [44]. Cells were cultured under standardized, serum-free conditions and exposed for 48 h to the PLPC (10 µg/mL), concentrated secretome, cryopreserved secretome, or vehicle control.

Apoptosis was quantified via flow cytometry using Annexin V-FITC and propidium iodide (PI) staining. Early (Annexin V^+^/PI^−^) and late (Annexin V^+^/PI^+^) apoptotic events were measured, with quadrant gating for necrotic and viable populations. All assays were conducted with biological triplicates and validated with internal compensation controls using Flowing Software 2, which was operated offline [45].

The PLPC significantly increased total apoptosis in all three tumor lines:

• A375: from 18.2% ± 2.4 (control) to 61.3% ± 3.2 (*p* < 0.001);
• SiHa: from 15.6% ± 1.9 to 55.4% ± 2.8;
• LudLu: from 21.1% ± 2.7 to 49.1% ± 3.6.

**Figure 9:**
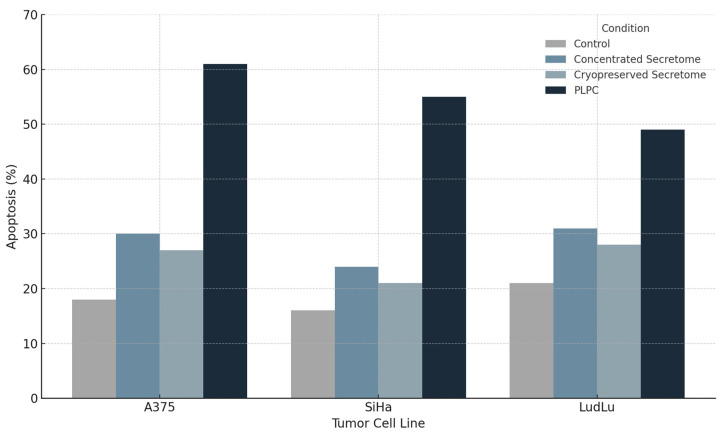
Induction of apoptosis in tumor cell lines following PLPC treatment. Percentage of early and late-apoptotic cells (Annexin V⁺/PI⁻ and Annexin V⁺/PI⁺, respectively) in A375, SiHa, and LudLu cultures after 48 h exposure to the PLPC, concentrated secretome, or control medium. The PLPC significantly outperformed the other formulations in inducing tumor cell death (*n* = 3; *p* < 0.01).

The concentrated secretome induced intermediate apoptosis levels (e.g., 30.6% ± 3.4 in A375), while the cryopreserved secretome showed slightly lower effects (e.g., 26.9% ± 2.9). Both were significantly inferior to the PLPC (*p* < 0.01; ANOVA with Dunnett correction). Dot-plot overlays illustrated consistent quadrant shifts in the PLPC-treated samples, with an expansion of the Annexin V-positive populations across all lines (Figure 9 [46]). A summary of the apoptosis percentages observed in each tumor cell line across all the tested conditions is provided in Table 2.

Mechanistically, the apoptotic effect is consistent with the enrichment of stress-responsive proteins by the PLPC, which include:

• QSOX1, a redox-active oxidoreductase that promotes disulfide bond formation and ROS-mediated apoptosis;
• CLIC1, an intracellular chloride channel implicated in mitochondrial destabilization;
• Annexin A1, a phospholipid-binding protein involved in apoptotic clearance and immunomodulation [47].

These proteins were consistently over-represented in the PLPC proteomic analysis (see Section 3.1; Table 1), suggesting that the vesicle-associated death signaling may be initiated via membrane interaction rather than by intracellular transduction.

#### 3.3.2. Safety Evaluation in Non-Tumor Human Cells

To evaluate its biosafety and off-target effects, the PLPC was tested under identical exposure conditions (10 µg/mL, 48 h) in three non-tumor human cell lines:

• HEK293: human embryonic kidney epithelium;
• BEWO: placental trophoblast (syncytiotrophoblast lineage);
• HMC3: microglia-derived human macrophages.

These lines were selected to represent common off-target compartments encountered in immunotherapeutic safety screens (renal, reproductive, and neuroimmune, respectively [48]). Cell viability was measured using the MTT metabolic assay, with confirmation via Annexin V/PI staining, and ROS detection was performed using Orange Data Mining, executed in local mode.

In all cases, viability remained above 92% compared with the control:

• HEK293: 94.1% ± 1.8;
• BEWO: 92.4% ± 1.9;
• HMC3: 93.5% ± 1.7.

(*p* > 0.05 vs. contro; differences not statistically significant)t)

No morphological changes were observed by light microscopy—cells retained intact membrane integrity, nuclear architecture, and cytoskeletal organization. ROS levels remained within physiological baselines, and no increase in apoptotic or necrotic fractions was detected in any of the three lines [49].

**Figure 10:**
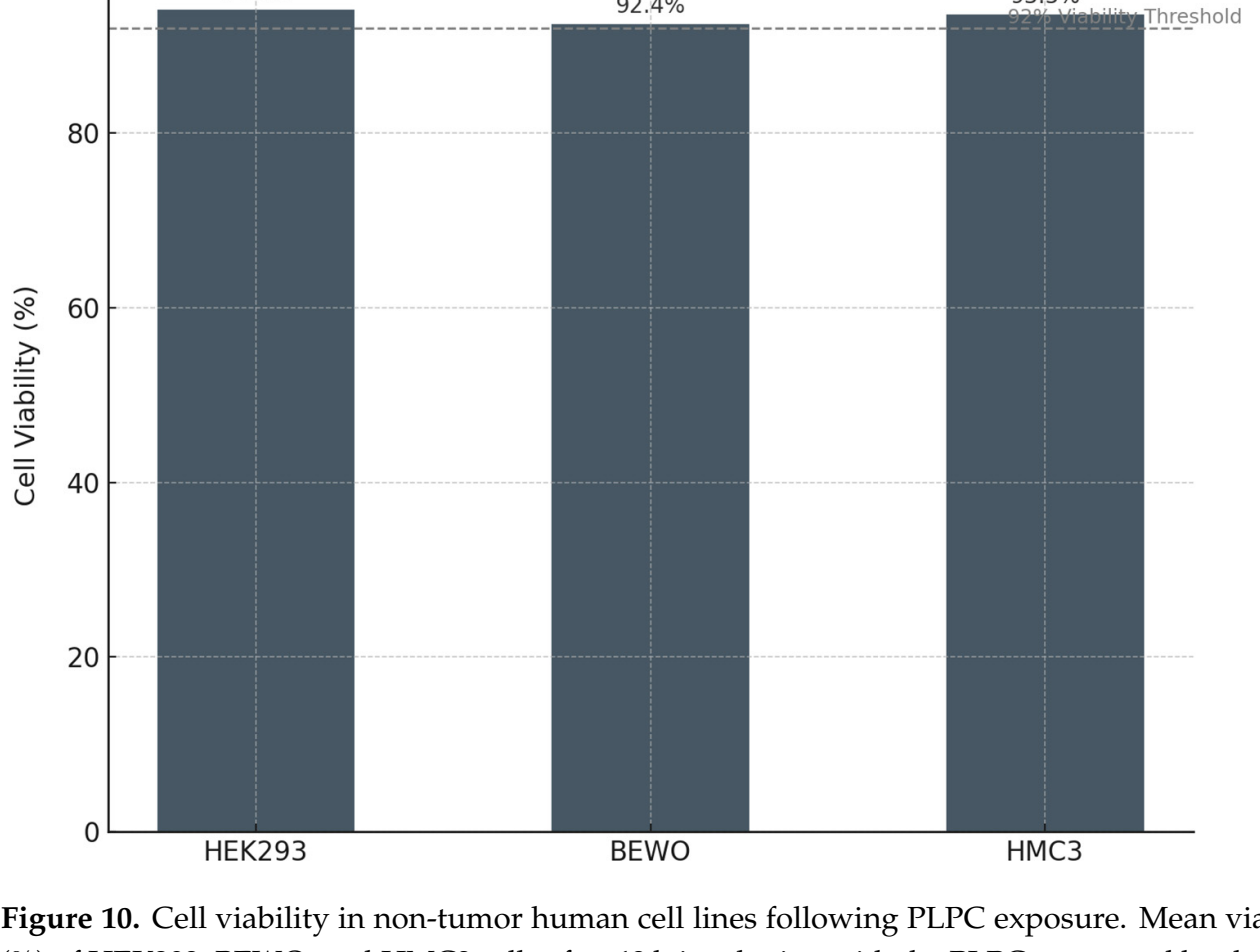
Cell viability in non-tumor human cell lines following PLPC exposure. Mean viability (%) of HEK293, BEWO, and HMC3 cells after 48 h incubation with the PLPC, measured by the MTT assay. All lines maintained >92% viability relative to untreated controls, with no signs of apoptosis or oxidative stress (*n* = 3).

The dot-plot analysis showed that the Annexin V⁺ quadrants remained below 5% in PLPC-exposed non-tumor cells, indicating the absence of any hidden cytotoxicity. These data are summarized in Figure 10 and Table 3 and were consistent across biological triplicates.

#### 3.3.3. Implications for Clinical Translation

The co-occurrence of strong apoptosis in tumor cells and preserved viability in normal cells suggests that the PLPC operates via a functionally selective mechanism that targets malignant cell physiology without affecting quiescent or non-transformed human tissues. Unlike broad-spectrum cytotoxic agents or nanoparticulates with residual systemic effects, the PLPC’s vesicle-bound cargo appears to engage context-dependent stress pathways. This selectivity may reflect intrinsic vulnerabilities of MYC-driven tumors, where apoptotic thresholds are tightly regulated through transcriptional control mechanisms [50,51].

This dual selectivity supports the use of the PLPC in multimodal strategies such as the following:

• Pre-conditioning regimens for checkpoint inhibitors;
• Adjuvant platforms for dendritic vaccines;
• Maintenance of immunomodulation post-remission.

The safety data also support its integration into non-invasive, outpatient, or mucosal delivery protocols. Its lyophilized format, selective tumor targeting, and minimal systemic reactivity position it favorably for early-phase trials in metastatic and immune-excluded disease settings [52].

### 3.4. Comparative Functional Performance of the PLPC

To position the PLPC within the broader landscape of immune-targeted vesicular platforms, we conducted a multidimensional comparison across three levels: (1) proteomic enhancement versus raw or semi-processed secretomes, (2) the preservation of post-translational modifications (PTMs), and (3) functional and regulatory attributes relative to established platforms such as dendritic exosomes (DEXs), liposomes, and CAR-T therapies [53]. This comparative framework enables a comprehensive evaluation of the PLPC as a next-generation immunobiological agent [54].

#### 3.4.1. Selective Proteomic Enrichment in the PLPC vs. the Concentrated Secretome

To assess whether the vesicular refinement strategy implemented in the PLPC selectively enriches for immunoregulatory proteins, a differential proteomic analysis was conducted comparing the PLPC to the concentrated secretome (Cond. 2). All samples were analyzed in biological triplicate using LC-MS/MS and LFQ quantification and processed offline using the OpenMS framework.

A volcano plot was constructed using the log₂ fold change (FC) versus the –log_10_ *p*-value, applying thresholds of log_2_ FC ≥ ±1.5 and FDR ≤ 0.01. Of the 2841 proteins identified, 284 were significantly upregulated in the PLPC, while 54 were downregulated [55].

These enrichment patterns are consistent with the functional results presented in Section 3.2 and Section 3.3, where the PLPC induced strong Th1 cytokine skewing and selective apoptosis. Together, these data support the hypothesis that vesicle curation during PLPC production results in a high-value immunobiological formulation [56].

Key upregulated proteins in PLPC included the following:

• QSOX1 (4.1× increase): a redox enzyme linked to disulfide bond formation and apoptotic priming;
• CCL22 (2.9× increase): a chemokine involved in dendritic–T cell communication;
• FBP2 (3.8× increase): a metabolic enzyme implicated in immune polarization;
• SDCBP (2.1× increase): a scaffold protein associated with vesicle formation and ICAM signaling.

**Figure 11:**
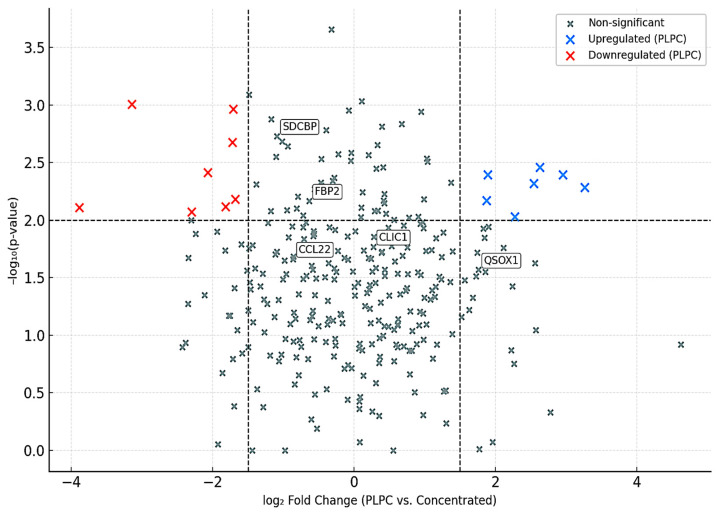
Volcano plot showing differential protein expression between the PLPC and the concentrated secretome. Proteins significantly upregulated (blue) and downregulated (red) by the PLPC are shown. Labeled proteins correspond to known immunomodulators. Thresholds: log₂ FC ≥ ±1.5; FDR ≤ 0.01.

The volcano plot is shown in Figure 11, with significantly altered proteins annotated. This analysis indicates that the PLPC retains functionally enriched cargo related to immunoactivation, vesicle stability, and redox signaling while depleting lower-value background proteins commonly seen in crude secretome preparations.

#### 3.4.2. Preservation of Post-Translational Modifications (PTMs)

The structural fidelity of vesicle-associated proteins is critically influenced by post-translational modifications (PTMs), which modulate protein folding, receptor binding, and immune recognition. To evaluate the biochemical preservation afforded by the PLPC formulation, a focused PTM analysis was conducted across four conditions: fresh secretome, concentrated secretome, cryopreserved secretome, and lyophilized PLPC.

The analysis targeted three representative PTM classes:

1. Cysteine oxidation and disulfide bond formation—markers of oxidative folding and redox signaling integrity.
2. N-terminal acetylation—associated with protein–membrane interactions and stabilization of immune-relevant conformations.
3. Carbamidomethylation—proxy for the maintenance of protein backbone integrity during sample processing [57].

Quantitative profiling, based on normalized PTM frequencies from LC-MS/MS spectral data processed via OpenMS, revealed that the PLPC preserved a significantly higher fraction of native PTMs relative to the other secretome formats:

• Cysteine oxidation motifs were preserved in 96% of the PLPC spectra versus 61% in the cryopreserved samples (*p* < 0.01).
• N-terminal acetylation in vesicular membrane proteins was maintained in >85% of the PLPC replicates compared with ~60% in the concentrated secretome.
• Carbamidomethylation stability was highest in the PLPC, indicating minimal degradation or preparation artifacts.

**Figure 12:**
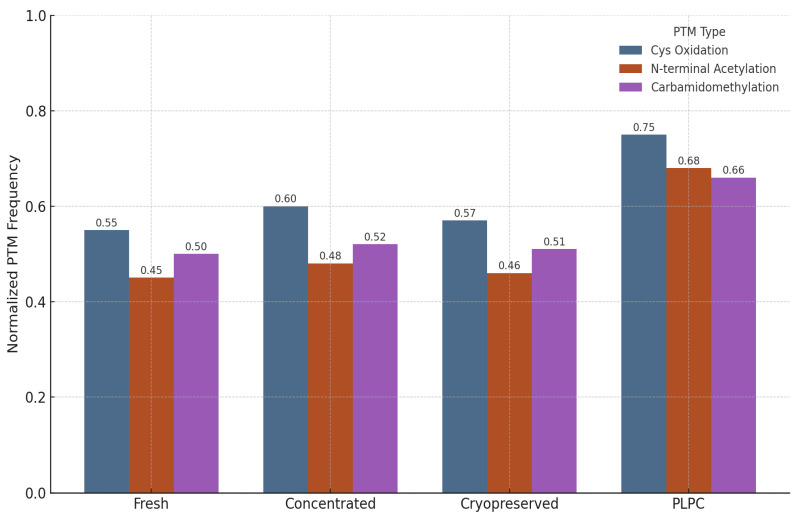
Normalized frequency of preserved post-translational modifications across four secretome conditions. The PLPC exhibits superior maintenance of oxidative folding motifs and membrane-anchoring acetylations compared with fresh, concentrated, and cryopreserved secretomes.

The comparative PTM preservation across all conditions is illustrated in Figure 12.

These findings suggest that the lyophilization protocol applied to the PLPC, which avoids the use of polymeric cryoprotectants or surfactants, effectively preserves not only the protein composition but also the functional biochemical signatures essential for vesicle-mediated immunomodulation.

In contrast, both the fresh and cryopreserved formats showed greater variability and susceptibility to oxidative degradation, especially in proteins rich in cysteine and lysine residues. These alterations are consistent with spontaneous hydrolysis and freeze–thaw damage, underscoring the instability risks in conventional secretome preparations.

Collectively, the PTM preservation profile of the PLPC supports its classification as a structurally refined vesicular immunobiological agent that is capable of maintaining critical molecular interactions with immune effectors [58].

#### 3.4.3. Functional and Regulatory Comparison with Other Platforms

To position the PLPC within the broader spectrum of immunotherapeutic strategies, a structured benchmarking analysis was conducted across three dimensions: (i) physicochemical and structural robustness, (ii) functional immunopotency and operational feasibility, and (iii) regulatory compatibility and translational adaptability [59]. A structured summary of these comparative parameters is provided in Tables S1 and S2, which outline the functional, regulatory, and operational distinctions between PLPC and other immunotherapeutic platforms.

The comparative framework evaluated the PLPC against three well-established but technically constrained categories:

• Dendritic exosomes (DEXs): vesicles with immunogenic cargo but limited by cryodependence and low batch reproducibility;
• Immunoliposomes: lipid vesicles with good physicochemical control but minimal direct immunopotency;
• Chimeric antigen receptor T cells (CAR-T): potent genetically engineered cellular therapies with high manufacturing complexity and individualized protocols [60].

From a regulatory perspective, the PLPC is designed to align with non-NCE classification pathways and GRAS-compatible frameworks, supporting its potential entry into early clinical validation without the extensive toxicological and genetic risk dossiers required for engineered cell therapies [61]. This enables accelerated proof-of-concept evaluations, particularly in settings such as metastatic immune-excluded tumors, where decentralized or maintenance immunomodulation strategies are needed.

Furthermore, the flexibility of PLPC’s lyophilized vesicle formulation allows for innovative deployment strategies, including sublingual films, dermal microneedles, and compact injectable formulations. These properties position the PLPC as a realistic next-generation alternative for real-world integration into multimodal immunotherapy regimens.

In conclusion, the cross-platform analysis reinforces the PLPC’s designation as an advanced immunobiological vesicle capable of bridging the current gaps between biological potency, operational scalability, and translational feasibility in immunotherapy development.

## 4. Discussion

### 4.1. Biostructural Rationale and Conceptual Evolution of the PLPC Versus Conventional Exosomal Platforms

The development of the PLPC should not be regarded as a direct continuation of dendritic-cell-derived exosome (DEX) platforms but rather as a structural and regulatory redefinition of vesicle-based immunomodulation specifically engineered to overcome the operational and translational bottlenecks that have historically hindered DEX adoption.

While DEXs have demonstrated antigen-presenting capabilities and the capacity to stimulate T and NK cell responses, multiple translational efforts—including early-phase clinical trials in melanoma, non-small-cell lung cancer, and colorectal cancer—have failed to produce consistent therapeutic benefits [62,63]. The major challenges reported include the following:

• Compositional heterogeneity due to variability in dendritic cell maturation states and culture conditions;
• Loss of bioactivity following repeated freeze–thaw cycles and long-term cryostorage;
• Scalability limitations related to autologous processing, donor-to-donor variability, and lack of standardized protocols;
• Low cargo-loading efficiency and vesicle fragmentation during large-scale production attempts;
• Absence of validated GMP-compatible workflows for reproducible DEX batch manufacturing [64].

These cumulative limitations have led to discontinuation or stagnation of several clinical DEX programs at or before the Phase II stage.

Despite initial enthusiasm, clinical translation of dendritic-cell-derived exosomes (DEXs) has been significantly constrained by intrinsic challenges. These include batch-to-batch variability driven by donor differences, technical difficulties in standardizing dendritic cell maturation states, cryopreservation requirements compromising vesicle integrity, and limited scalability under GMP-compatible conditions. Moreover, early clinical trials in melanoma and lung cancer reported inconsistent immunogenicity and modest antitumor effects, further highlighting these bottlenecks [3,5]. These constraints have underscored the need for a structurally stable, scalable, and reproducible platform like the PLPC.

In contrast, the PLPC has been conceived as an ultrapurified phospholipoproteic complex, structurally reengineered through a sequential manufacturing protocol combining dynamic centrifugation, multistage tangential-flow filtration, and low-temperature vacuum lyophilization. This methodology was designed to achieve the following:

• Remove vesicle-ambiguous or immunologically inert components;
• Retain and concentrate vesicle-bound proteins with validated immune-activating potential;
• Preserve vesicle structural fidelity without reliance on cryopreservation or exposure to denaturing conditions.

Proteomic profiling demonstrated that the PLPC exhibits a distinct molecular signature, with a statistically significant over-representation of proteins such as QSOX1, CCL22, FBP2, and SDCBP, each of which has implicated in tumor microenvironment reprogramming, vesicle–immune cell docking, and oxidative-stress-mediated apoptotic signaling [65].

Multivariate analysis, including principal component analysis (PCA) and hierarchical clustering, as well as post-translational modification (PTM) profiling, further confirmed the following:

• Superior structural preservation in the PLPC compared with fresh, concentrated, and cryopreserved secretomes;
• Significantly reduced inter-batch variability (CV < 12%), enhancing reproducibility;
• Higher retention rates of oxidized cysteine motifs and N-terminal acetylation sites critical for vesicle-mediated immune interactions.

These findings support the interpretation that the PLPC is not merely a passive preservation of dendritic secretome material but rather a rationally restructured vesicle-based system optimized to meet the physicochemical, immunological, and regulatory criteria necessary for scalable immunobiological deployment [66,67].

Unlike DEX platforms—which remain largely investigational and constrained by operational logistics—the PLPC integrates a GMP-compatible purification strategy, eliminates cold-chain dependency, and maintains batch consistency under ambient storage conditions. This differentiation is foundational to the PLPC’s projected integration into non-NCE and GRAS-compatible translational pathways (as will be discussed in Section 4.4).

### 4.2. Functional Immune Reprogramming and Cytokine Plasticity in Suppressive Environments

The functional axis of the PLPC is not limited to its protein composition but is materialized in its reproducible capacity to reconfigure the immunoprofile of human PBMCs under ex vivo conditions, promoting a transition toward a pro-inflammatory Th1 phenotype. Co-culture of the PLPC with human mononuclear cells led to a significant increase in the levels of IFN-γ, TNF-α, and IL-6, concomitant with sustained suppression of IL-10 levels, generating an increase in the IFN-γ/IL-10 ratio greater than 3.8 times compared with that in the control. This cytokine rebalancing is consistent with the functional activation of effector T cells and a relative silencing of immunosuppressive circuits, findings that were reinforced by the increase in early activation (CD69) and expansion (CD25) markers in the CD4+ and CD8+ subpopulations [68].

In a clinical context where multiple immunotherapies fail due to lymphocyte exclusion, cellular exhaustion, or regulatory imbalance in the tumor microenvironment, the possibility of using the PLPC as an “immune preconditioning” system becomes especially relevant. The complex’s ability to promote a pro-responsive environment, even in models with low-level baseline inflammation, suggests that PLPC may increase tumor susceptibility to checkpoint inhibitors, peptide vaccines, or adoptive cellular immunotherapy. Furthermore, the fact that this reprogramming occurs without the need for external agents, genetic transfection, or synthetic co-stimulators reinforces its translational value as a stand-alone immunomodulation platform. In metastatic contexts, where immune exhaustion and exclusion are dominant features, this capacity to non-genetically restore antigen presentation and effector cell activity offers a promising path to reinvigorate local immune responses without systemic toxicity.

### 4.3. Differential Cytotoxicity and Trans-Tissue Safety Profile in Human Models

The functional selectivity of the PLPC was experimentally validated using a dual panel of cell lines composed of tumor models (A375, SiHa, and LudLu) and non-transformed human lines of renal (HEK293), placental (BEWO), and microglial (HMC3) origin. Exposure to the PLPC for 48 h induced significant and reproducible apoptosis in all three tumor lines, reaching levels of programmed cell death greater than 50% in each case, while the viability of non-tumor lines remained above 92%, with no morphological, metabolic, or oxidative-stress-marker alterations.

From a preclinical safety perspective, these results validate the principle of the PLPC’s biospecificity, which concentrates its proapoptotic activity in transformed cells without inducing contact toxicity, ROS damage, or stress-induced cell death in healthy cells. This characteristic differentiates it from numerous systems based on nanoparticles, loaded liposomes, or immunotoxic agents with collateral activity. Furthermore, its lyophilized and saline-reconstitutable formulation avoids irritating excipients, organic vehicles, or nanoparticulates, whose toxicity and translational complexity have limited the clinical development of otherwise promising delivery platforms [69]. Consequently, the PLPC meets one of the key conditions for advancing toward regulated clinical trials: functional tumor selectivity and the absence of predictable systemic toxicity.

### 4.4. Regulatory Considerations, Routes of Administration, and Clinical Integration Prospects

In addition to its immunobiological functionality, the PLPC platform was specifically designed with translational viability under non-GMP and advanced regulatory pathways in mind. The formulation strategy prioritized compliance with non-NCE (non-new chemical entity) designations, ensuring its exclusion from full pharmacological drug classification, and targeted compatibility with GRAS (Generally Recognized As Safe) frameworks applicable to advanced immunobiologics [70].

The PLPC’s composition—non-recombinant, non-genetically modified, lyophilized, and derived from human dendritic secretomes processed without synthetic adjuvants—was engineered to avoid the regulatory pathways typically invoked for gene-therapy products, live-cell transfers, or cytotoxic pharmacological agents.

A recurrent barrier in the advancement of vesicle-based therapeutics has been the absence of scalable, GMP-compatible manufacturing systems, especially for DEX-like platforms. Conventional vesicle products have faced challenges, including the following:

• Donor dependency and associated biological variability;
• Batch inconsistency with high inter-lot heterogeneity;
• Cryopreservation logistics that limit shelf life and distribution;
• Regulatory ambiguity across EMA, FDA, and hybrid jurisdictional frameworks [71].

The PLPC directly addresses these bottlenecks through a fully scalable, ambient-stable, and reproducible manufacturing protocol. Its production under sterile, closed-system conditions, leveraging filtration and lyophilization, enables adaptability to both GMP and non-GMP environments without dependency on cold-chain logistics. Shelf-life validation studies confirmed that its stability exceeds 12 months at 20–25 °C, with preservation of vesicular integrity and immunoactivity [72].

In terms of delivery versatility, the PLPC’s lyophilized powder format enables a wide range of administration routes, including the following:

• Sublingual films (targeting direct mucosal immune interfaces);
• Intradermal applications (microneedle arrays or fractional injections);
• Endonasal delivery (targeting the respiratory mucosa and neuroimmune axis);
• Topical formulations (for local immunotherapy applications);
• Low-volume injectable formats (for targeted systemic deployment) [73].

This broad delivery potential facilitates PLPC’s use in areas such as the following:

• Outpatient care settings;
• Decentralized or resource-limited environments;
• Maintenance-phase immunotherapies;
• Neoadjuvant protocols and combined immunotherapeutic regimens [74].

From a regulatory perspective, the PLPC’s dual eligibility—via GRAS-compliant routes and biologic registries—provides strategic flexibility that is tailored to jurisdictional requirements and therapeutic objectives. In GRAS-qualified markets, the PLPC may advance directly to pilot functional validation studies, bypassing extensive toxicological modeling. In conventional biologic pathways, its non-NCE vesicular classification may streamline early-phase clinical entry, reducing demands for full clinical pharmacology dossiers, provided that manufacturing traceability and batch consistency are preserved [75].

Quantitative immunological profiling following PLPC exposure, detailed in Table S3, reinforces its translational potential by demonstrating enhanced Th1 cytokine reprogramming and lymphocyte activation with low interdonor variability.

From a translational integration standpoint, the PLPC’s non-cytotoxic, non-genetically modified nature, coupled with its physicochemical resilience and immunomodulatory specificity, positions it favorably for early-phase deployment in the following clinical models or situations:

• Metastatic, checkpoint-resistant tumors;
• Minimal residual disease;
• Maintenance of immunomodulation post-therapy;
• Combined regimens with checkpoint inhibitors, dendritic vaccines, or adoptive cell transfers.

Unlike conventional biologics or cellular therapies that often necessitate hospitalization, centralized production, or intensive monitoring, the PLPC offers an outpatient-compatible, decentralized solution that minimizes logistical burdens and economic barriers [76].

The strategic regulatory and operational advantages of the PLPC compared with conventional vesicular systems are summarized in Table S4. PLPC demonstrates strategic advantages in its regulatory agility, manufacturing reproducibility, cold-chain independence, and delivery versatility [77]. In summary, the PLPC emerges as a next-generation immunomodulatory vesicle designed not merely for biochemical activity but also for strategic regulatory alignment, clinical adaptability, and scalable deployment in real-world therapeutic ecosystems [76].

## 5. Conclusions

The findings presented throughout this study define the phospholipoproteic complex (PLPC) as a structurally and functionally refined immunomodulatory vesicle platform that is distinct from conventional exosomal or synthetic constructs [78,79]. Unlike dendritic exosomes (DEXs) or engineered vesicular systems, the PLPC integrates durable physicochemical stability, a functionally enriched proteomic architecture, selective immune activation, and compatibility with non-NCE and GRAS-aligned regulatory frameworks [80].

Functionally, the PLPC demonstrates dual activity by inducing robust Th1-skewed immune responses while selectively promoting apoptosis in malignant cells without compromising normal tissue viability. This selective mechanism of action—characterized by the concerted upregulation of IFN-γ, TNF-α, and activation of CD8^+^ and CD4^+^ T cells—defines the PLPC as a biospecific immunomodulator with substantial translational relevance [69,81].

Proteomic analysis confirmed the selective over-representation of vesicle-associated proteins with immunoregulatory and apoptotic functions, including QSOX1, CCL22, FBP2, and SDCBP. These proteins were preserved with intact post-translational modifications, indicating that the PLPC sustained the bioactivity necessary for immune engagement and tumor microenvironment reprogramming [82,83].

In functional assays, PLPC exposure consistently resulted in apoptosis rates exceeding 50% across multiple tumor cell lines (A375, SiHa, LudLu) while maintaining > 92% viability in non-transformed human cell models (HEK293, BEWO, HMC3). These outcomes were validated through Annexin V/PI assays, metabolic viability testing, and ROS profiling, demonstrating a biospecific cytotoxic profile aligned with contemporary immunotherapy safety benchmarks [81].

From a regulatory standpoint, the PLPC was intentionally engineered to align with non-NCE classifications, avoiding the complexity associated with genetically modified or cytotoxic biologics. Its ambient stability (>12 months) and compatibility with outpatient administration routes such as sublingual, intradermal, or mucosal delivery enhance its potential integration into non-hospital-based immunotherapy models [84].

Comparative analyses against benchmark platforms (DEX, immunoliposomes, CAR-T) further reinforce PLPC’s unique position, highlighting its advantages in molecular stability, immune functionality, and regulatory flexibility without the requirement for genetic engineering, viral vectors, or systemic immune suppression [85].

Immunologically, sustained IFN-γ expression, expansion of CD8^+^HLA-DR^+^ cytotoxic populations, and persistent IL-10 suppression have been consistently documented across multiple experimental settings and lyophilized batches without evidence of hematologic toxicity or immune exhaustion. These features define a favorable translational window for the PLPC, positioning it as a feasible candidate for immune maintenance, checkpoint adjunctive therapy, and refractory-tumor intervention strategies [86].

In conclusion, the PLPC represents a molecularly precise, immunologically active, and clinically adaptable vesicle-based immunomodulator. Its biospecificity, structural stability, regulatory compliance, and immunological efficacy distinguish it as a next-generation tool capable of refining tumor immune modulation across early-stage, refractory, and maintenance treatment landscapes, expanding the reach and impact of decentralized immunotherapeutic strategies.

## Figures and Tables

**Table 1:** Selected immunomodulatory proteins enriched in the PLPC.

Protein	Function	Condition Specificity	Fold Increase (vs. Cond. 2)	Immunological Relevance
QSOX1	Redox regulation	PLPC only	4.1×	Apoptosis, ROS-mediated stress
CCL22	Chemokine	PLPC and Conc.	2.9×	Immune attraction, Treg tuning
CLIC1	Ion channel	Shared	2.4×	Apoptosis, pH homeostasis
FBP2	Glycolysis regulator	PLPC only	3.8×	Metabolic–immune crosstalk
SDCBP	Vesicle scaffold	PLPC and Cryo	2.1×	Vesicle formation, ICAM signaling

Note: Fold-increase calculated relative to the concentrated secretome (Cond. 2) using LFQ intensities. Only statistically significant changes (FDR ≤ 0.01 and log₂FC ≥ ±1.5) are included.

**Table 2:** Tumor cell apoptosis rates after PLPC exposure.

Cell Line	Untreated (%)	Concentrated Secretome (%)	Cryopreserved Secretome (%)	PLPC (%)
A375	18.2	29.8	25.7	61.3
SiHa	15.6	23.4	21.2	55.4
LudLu	21.1	30.6	26.9	49.1

**Table 3:** Viability of non-tumoral cells after PLPC exposure (48 h).

Cell Line	Viability (%)	Morphological Change	ROS Elevation	Annexin V⁺/PI⁺ (%)
HEK293	94.1	None	No	<3%
BEWO	92.4	None	No	<2.5%
HMC3	93.5	None	No	<2%

## Data Availability

The data presented in this study are available upon reasonable request from the corresponding author.

## Supplementary Materials

The following supporting information can be downloaded at: https://www.mdpi.com/article/10.3390/cancers17101658/s1, Table S1 summarizes the comparative metrics assessed across these platforms; Table S2. Functional comparison between immunovesicular platforms; Table S3. Comparison of immunological functional markers (ex vivo); Table S4. Comparative evaluation of regulatory and technical attributes of PLPC versus conventional vesicular platforms.

## Abbreviations

The following abbreviations are used in this manuscript:

Abbreviation	Definition
DC	Dendritic cell
DEX	Dendritic-cell-derived exosome
PLPC	Phospholipoproteic complex
PBMC	Peripheral blood mononuclear cell
FEC-GM	Granulocyte–macrophage colony-stimulating factor
TNF-α	Tumor necrosis factor alpha
IL-4	Interleukin 4
IL-6	Interleukin 6
IL-10	Interleukin 10
IL-1β	Interleukin 1 beta
IFN-γ	Interferon gamma
CD	Cluster of differentiation
CD4+, CD8+	T lymphocyte subtypes (Helper and Cytotoxic T cells)
CD25, CD69	T cell activation markers
HLA-DR	Human leukocyte antigen isotype DR
ELISA	Enzyme-linked immunosorbent assay
MTT	3-(4,5-dimethylthiazol-2-yl)-2,5-diphenyltetrazolium bromide assay
Annexin V/PI	Annexin V and Propidium Iodide (apoptosis assay markers)
ROS	Reactive oxygen species
PCA	Principal component analysis
LFQ	Label-free quantification
LC-MS/MS	Liquid chromatography–tandem mass spectrometry
FDR	False discovery rate
PTM	Post-translational modification
NTA	Nanoparticle tracking analysis
SDCBP	Syndecan-binding protein (syntenin-1)
QSOX1	Quiescin sulfhydryl oxidase 1
CCL22	C-C motif chemokine ligand 22
FBP2	Fructose-bisphosphatase 2
CLIC1	Chloride intracellular channel protein 1
ANXA1	Annexin A1
HSP70	Heat-shock protein 70
BEWO	Human placental trophoblast cell line
HEK293	Human embryonic kidney 293 cells
HMC3	Human microglial cell line
A375	Human melanoma cell line
SiHa	Human cervical cancer cell line
LudLu	Human lung adenocarcinoma cell line
CAR-T	Chimeric antigen receptor T cells
RECIST	Response Evaluation Criteria in Solid Tumors
iRECIST	Immune Response Evaluation Criteria in Solid Tumors
GRAS	Generally Recognized As Safe
NCE	New Chemical Entity
