# Supplementary material for: Beyond Exosomes: An Ultrapurified Phospholipoproteic Complex (PLPC) as a Scalable Immunomodulatory Platform for Reprogramming Immune Suppression in Metastatic Cancer
Source: Cancers (Basel). 2025 May 14;17(10):1658. doi: 10.3390/cancers17101658 (PMC12110133; doi:10.3390/cancers17101658)
Supplement: Supplementary file 1 [file cancers-17-01658-s001.zip › cancers-3588167-supplementary.pdf]

Table S1 summarizes the comparative metrics assessed across these platforms.

Parameter	DEX	Liposomes	CAR-T Cells	PLPC
Stability	Low	High	Moderate	High
Cryopreservation	Required	No	Yes	No
Batch Consistency	Variable	High	Low	High
Tumor Apoptosis	Indirect	None	Yes	Yes
Regulatory Classification	Complex	Approved	Complex	GRAS-compatible / Non-NCE
Cost of Production	High	Medium	Very High	Low–Medium
Delivery Versatility	Injection only	Versatile	IV only	Sublingual, endonasal, intradermal

Table S1 provides a structured multidimensional comparison of critical functional and regulatory parameters between PLPC and established immunotherapeutic platforms, including dendritic cell-derived exosomes (DEX), immunoactive liposomes, and CAR-T cell therapies.

Regarding structural resilience, PLPC exhibits high ambient stability confirmed for over 12 months without cryopreservation, surpassing the low stability of DEX and equaling or exceeding that of engineered liposomes. This robust stability profile stems from PLPC's lyophilized preparation, which preserves vesicular architecture without the need for synthetic cryoprotectants or specialized cold chain logistics.

In terms of batch consistency, PLPC benefits from centralized, standardized manufacturing workflows, resulting in minimal inter-batch variability. This addresses a longstanding limitation of DEX, where donor dependency and culture conditions generate high heterogeneity. By contrast, CAR-T therapies, while potent, suffer from low batch consistency due to the individualized nature of patient-derived manufacturing.

Functionally, PLPC demonstrates direct immunomodulatory activity, with ex vivo evidence of Th1 polarization, cytokine modulation, and selective tumor cell apoptosis. These functional outputs position PLPC at the same potency level as CAR-T cells, but without the genetic engineering, viral transduction, or systemic inflammatory risks commonly associated with cell-based therapies. Liposomes, despite their structural versatility, generally lack intrinsic immunomodulatory properties unless artificially loaded with immune adjuvants.

Importantly, PLPC exhibits tumor apoptosis induction validated across multiple tumor models, similar to CAR-T therapies but achieved via vesicle-mediated biological reprogramming rather than through cytotoxic genetic constructs.

From a regulatory perspective, PLPC is designed to fit within GRAS-compliant and non-NCE pathways, unlike DEX and CAR-T therapies that face complex regulatory classifications due to their biological source variability or genetic engineering components. This strategic alignment enhances the feasibility of PLPC's integration into early-phase clinical pipelines.

Regarding delivery modalities, PLPC's lyophilized vesicle format enables a wide range of administration routes—including sublingual, endonasal, intradermal, and parenteral—unlike the injection-only routes generally required for DEX and CAR-T therapies. This flexibility expands the possibilities for decentralized, outpatient, or maintenance immunotherapy programs, particularly in resource-limited settings.

While estimated production costs are included for context, it is critical to emphasize that the comparative analysis primarily focuses on biological robustness, regulatory adaptability, and functional immunopotency, not on economic projections.

Overall, Table S1 underscores PLPC's position as a structurally stable, immunologically active, regulatorily streamlined, and operationally versatile vesicular platform designed to overcome the primary limitations of conventional exosome and CAR-T cell therapies.

Table S2. *Functional comparison between immunovesicular platforms.*

Comparison of critical performance parameters between PLPC and conventional vesicular or cellular systems. PLPC exhibits superior ambient stability, batch reproducibility, absence of cryopreservation requirements, and administration versatility, with a regulatory profile supporting both GRAS and non-NCE classifications.

Key parameter	DEX	Liposomes immunoactive	CAR-T Cells	PLPC
Structural stability	Low	High	Moderate	High
Requires cryopreservation	Yes	No	Yes	No
Direct immunomodulatory activity	Moderate	Null	High	High
Selective induction of tumor apoptosis	Hint	Null	High	High (validated on multiple lines)
Inter-batch reproducibility	Variable	High	Low	High
Industrial scalability	Limited	High	Very limited	High
Estimated cost per treatment (USD)	High (>10,000)	Medium (200–500)	Very high (>300,000)	Low-Medium (estimated 200–1,000)
Route of administration	Subcutaneous/IV injection	Multiple	Intravenous	Intradermal / Endonasal / Sublingual / IV
Current regulatory status	Phase I/II (isolated cases)	Approved (non-immune)	Approved in select oncology	GRAS-compliant / non-NCE

Key parameter	DEX	Liposomes immunoactive	CAR-T Cells	PLPC
Structural stability	Low	High	Moderate	High
Requires cryopreservation	Yes	No	Yes	No
Direct immunomodulatory activity	Moderate	Null	High	High
Selective induction of tumor apoptosis	Hint	Null	High	High (validated on multiple lines)
Inter-batch reproducibility	Variable	High	Low	High
Industrial scalability	Limited	High	Very limited	High
Estimated cost per treatment (USD)	High (>10,000)	Medium (200–500)	Very high (>300,000)	Low-Medium (estimated 200–1,000)
Route of administration	Subcutaneous/IV injection	Multiple	Intravenous	Intradermal / Endonasal / Sublingual / IV
Current regulatory status	Phase I/II (isolated cases)	Approved (non-immune)	Approved in select oncology	GRAS-compliant / non-NCE

Table S2 presents a comprehensive comparative evaluation of critical functional parameters across major immunovesicular platforms, specifically highlighting the distinctive advantages of PLPC relative to dendritic cell-derived exosomes (DEX), immunoactive liposomes, and chimeric antigen receptor T cell (CAR-T) therapies.

Structurally, PLPC demonstrates high stability under ambient conditions (>12 months), a marked improvement over conventional DEX, which require strict cryogenic preservation, and surpassing CAR-T cells, which also demand complex storage infrastructures. This ambient resilience stems from PLPC's lyophilized architecture, enabling room-temperature stability without cryoprotectants or structural destabilization.

In terms of functional immunopotency, PLPC exhibits direct Th1-skewed immunomodulatory activity and selective tumor apoptosis induction validated across multiple tumor lines, matching or exceeding the functional efficacy of CAR-T cells, but without the complexity of genetic engineering. Conversely, liposomes, while structurally robust, exhibit minimal innate immunomodulatory activity, often requiring co-encapsulation of immunostimulants.

Manufacturing reproducibility is another critical distinction. PLPC achieves high batch-to-batch consistency through centralized, standardized production workflows, addressing the donor-dependency and inter-batch variability that historically limited DEX scalability. CAR-T therapies, while potent, remain individualized, patient-specific products with high variability and logistical complexity.

Scalability is a decisive parameter for translational feasibility. Unlike DEX, whose autologous nature restricts large-scale deployment, or CAR-T cells, which require individualized GMP manufacturing, PLPC's lyophilized, off-the-shelf format supports industrial scalability without compromising functional integrity.

Although Table S2 includes an estimation of production cost ranges, it is crucial to emphasize that the primary comparative focus remains biological and operational. The figures illustrate the feasibility of PLPC integration into real-world clinical settings through reduced logistical demands (e.g., no cold chain requirements) and versatile routes of administration.

Specifically, PLPC's administration options—including intradermal, endonasal, sublingual, and intravenous pathways—extend beyond the traditional intravenous-only delivery models seen in DEX and CAR-T cells, offering opportunities for decentralized, outpatient-compatible immunotherapy protocols.

From a regulatory perspective, PLPC is positioned under a GRAS-compliant and non-NCE classification framework, distinguishing it from DEX (largely experimental Phase I/II) and CAR-T therapies (classified under advanced therapeutic medicinal products requiring extensive regulatory dossiers).

Collectively, Table S2 underscores that PLPC was rationally engineered not merely to mimic exosomal functions, but to integrate enhanced structural resilience, functional immunopotency, manufacturing reproducibility, translational scalability, and regulatory agility within a single vesicular platform, positioning it for efficient and accessible clinical implementation.

Table S3. Comparison of immunological functional markers (ex vivo).

Quantitative evaluation of cytokine profiles and lymphocyte activation markers in human PBMCs following exposure to PLPC, compared to concentrated and cryopreserved secretomes and controls. PLPC exhibits the highest IFN-γ/IL-10 ratio and strongest lymphocyte activation, with superior interdonor consistency.

Marker / Indicator	Control	Concentrated	Cryo Secretome	PLPC	Functional interpretation
IFN-γ (pg / mL)	42.1	69.8	60.4	131.2	↑↑ Clear Th1 potentiation
IL-10 (pg / mL)	28.3	22.1	24.9	9.6	↓↓ Immunoregulatory suppression
IFN-γ / IL-10 ratio	1.49	3.16	2.42	13.67	Active immune reprogramming
% CD8+CD69+ (early activation)	6.4	11.9	10.1	26.3	Effective lymphocyte activation
% CD4+CD25+ (pre-expansion)	8.1	12.3	11.7	21.5	Functional preparation for specific proliferation
Interdonor variability (CV%)	23%	19%	28%	8%	High consistency of PLPC

Table S3 provides a quantitative evaluation of key immunological functional markers measured ex vivo in human PBMC cultures exposed to PLPC, compared to concentrated and cryopreserved secretomes and vehicle controls. The data demonstrate that PLPC elicits a markedly stronger Th1-polarized immune response, as evidenced by the highest IFN-γ concentrations (131.2 pg/mL) and the lowest IL-10 levels (9.6 pg/mL) among all tested conditions. The resulting IFN-γ/IL-10 ratio (13.67) reflects a significant reprogramming towards pro-inflammatory immune dominance, contrasting sharply with control and comparator groups. In parallel, PLPC-treated cultures exhibited substantial enhancement in early T cell activation, with $CD8^+CD69^+$ frequencies reaching 26.3% and $CD4^+CD25^+$ frequencies reaching 21.5%, indicative of both cytotoxic priming and helper T cell functional preparation. Notably, PLPC exposure achieved the lowest interdonor variability (CV 8%) compared to concentrated (19%), cryopreserved (28%), and control (23%) conditions, underscoring its reproducibility and robustness across different biological samples. Collectively, these findings substantiate PLPC's capacity to drive potent, consistent immune reprogramming without reliance on external adjuvants or genetic modifications, reinforcing its potential for translational immunotherapy applications.

Table S4. Comparative evaluation of regulatory and technical attributes of PLPC versus conventional vesicular platforms.

Regulatory or Technical Criteria	PLPC	Conventional Vesicular Systems
Regulatory classification (NCE status)	Non-NCE (no pharmacological status required)	Frequently classified as NCE
Genetic modification / viral transduction	Absent	Present in CAR-EVs or engineered platforms
Presence of animal-derived components	Not present	Frequently found in serum-based or tumor-derived vesicles
Stability at room temperature	Stable (>12 months confirmed)	Requires cryopreservation or −80°C storage
Manufacturing reproducibility	Centralized, standardized, batch-consistent	Donor-dependent, inter-batch variability
Routes of administration	Sublingual, topical, intradermal, parenteral	Mostly intravenous

Table S4 provides a comparative evaluation of key regulatory and technical attributes distinguishing the PLPC platform from conventional vesicular systems. Notably, PLPC is engineered as a non-NCE (non-new chemical entity) biologic, exempt from pharmacological drug classification requirements that typically constrain vesicle-based therapeutics. Unlike many conventional vesicular systems, particularly those derived from genetically modified cells or incorporating viral transduction methods, PLPC is entirely free of genetic manipulation and animal-derived components, enhancing its regulatory simplicity and biosafety profile. Its ambient stability—validated for over 12 months without the need for cryopreservation—contrasts sharply with the strict cold chain dependency observed in traditional exosomal platforms. In terms of manufacturing, PLPC achieves batch-to-batch consistency through centralized, standardized production workflows, mitigating the inter-donor and inter-lot variability that has historically limited the scalability of vesicle-based therapies. Furthermore, PLPC's lyophilized format enables versatile administration routes—including sublingual, topical, intradermal, and parenteral applications—offering operational flexibility absent in most intravenous-only vesicular systems. Collectively, these attributes position PLPC as a strategically engineered immunobiological platform optimized for real-world clinical integration under both GRAS-compatible and non-NCE regulatory pathways.
